# Sperm intrusion into the implantation-stage blastocyst and its potential biological significance

**DOI:** 10.1093/emph/eoad043

**Published:** 2023-12-23

**Authors:** Jayasree Sengupta, Thomas Kroneis, Amy M Boddy, Rahul Roy, Anish Sarkar, Deepayan Sarkar, Debabrata Ghosh, Berthold Huppertz

**Affiliations:** Department of Physiology, All India Institute of Medical Sciences, New Delhi, India; Division of Cell Biology, Histology & Embryology, Gottfried Schatz Research Center, Medical University of Graz, Graz, Austria; Department of Anthropology, University of California, Santa Barbara, Santa Barbara, CA, USA; Theoretical Statistics and Mathematics Unit, Indian Statistical Institute, New Delhi, India; Theoretical Statistics and Mathematics Unit, Indian Statistical Institute, New Delhi, India; Theoretical Statistics and Mathematics Unit, Indian Statistical Institute, New Delhi, India; Department of Physiology, All India Institute of Medical Sciences, New Delhi, India; Division of Cell Biology, Histology & Embryology, Gottfried Schatz Research Center, Medical University of Graz, Graz, Austria

**Keywords:** zona-free blastocyst, mid-luteal phase, sperm competition, trophectoderm, ultrastructure, microchimerism

## Abstract

The human embryo derives from fusion of oocyte and sperm, undergoes growth and differentiation, resulting in a blastocyst. To initiate implantation, the blastocyst hatches from the zona pellucida, allowing access from external inputs. Modelling of uterine sperm distribution indicates that 200–5000 sperm cells may reach the implantation-stage blastocyst following natural coitus. We show ultrastructural evidence of sperm cells intruding into trophectoderm cells of zona-free blastocysts obtained from the uterus of rhesus monkeys. Interaction between additional sperm and zona-free blastocyst could be an evolutionary feature yielding adaptive processes influencing the developmental fate of embryos. This process bears potential implications in pregnancy success, sperm competition and human health.

## INTRODUCTION

After successful fertilization in humans and non-human primates, the zygote modifies its extracellular covering, the zona pellucida, to restrict entry of additional sperm. Within the uterus, the embryo starts cavitation and develops into a blastocyst by differentiating into the placenta-specific trophectoderm and the inner cell mass. The blastocyst then hatches from its zona pellucida, initiates the implantation process, and thus provides a time window between hatching and implantation when the blastocyst remains without its protective zonal covering. Hatching allows the zona-free blastocyst to appose, adhere and attach to the maternal endometrial epithelium, triggering the process of implantation that shortly after results in maternal endometrium fully covering the implanting embryo.

During the period when the implantation-ready blastocyst is not protected from external inputs by its zona pellucida, supernumerary sperm either from peri-fertilization pool or from mating during the luteal phase may reach such zona-free blastocysts. Here, we present the first data supporting this possibility and discuss the potential physiological consequences of the intrusion of sperm into the zona-free implantation stage blastocyst in a natural conception cycle.

## MATERIALS AND METHODS

### Blastocyst retrieval and transmission electron microscopy

Nine archival samples of peri-implantation stage blastocysts were obtained from fertile female rhesus monkeys who cohabited with fertile males, samples derived as bi-products from our previous studies [1–3]. Twenty-one successfully mated female monkeys were laparotomized under anaesthesia (12 mg ketamine/kg body weight) on days 6–8 after ovulation. Their entire reproductive tracts were flushed and the flushings were examined under a stereozoom microscope to retrieve peri-implantation stage blastocysts. Fourteen blastocysts were obtained from 14 separate females; of which 5 embryos showed abnormalities, desynchrony or damage. The remaining nine synchronous zona-free blastocysts were selected for ultrastructural studies and fixed in 3% glutaraldehyde in 0.1 mol/l phosphate buffer (pH 7.2) overnight at 4°C, washed in cold phosphate buffer and post-fixed in 1% osmium tetroxide for 30 min, followed by dehydration in graded alcohol, and embedded in Spurr’s resin. The procedural details were previously described [2].

### Biophysical modelling assessing the number of potential sperm-blastocyst contacts

Modelling #1: Assumptions made for modelling #1 are (i) sperm cells are uniformly distributed along the length of the uterine lumen, and (ii) each unit behaves independently. The number of sperm cells entering is ‘*N*’, the length and diameter of the lumen are ‘ ℓ ’ and ‘*d*’, respectively, and the diameter of the implantation site is ‘*δ*’. The random number of sperm cells reaching the implantation site follows a binomial distribution with the parameters *N* and *δ*^2^/(4*d·*ℓ*).* Thus, the expected number of sperm cells reaching the implantation site is *Nδ*^2^/(4*d·*ℓ).

Modelling #2: Assuming the number of sperm cells decreases exponentially with distance, the modelling of sperm distribution may be parametrized by the proportion *p* of sperm cells that reach a distance ℓ−δ or more. In probabilistic terms, if the random distance *X* travelled by a sperm is assumed to be exponentially distributed with the rate parameter γ, we can obtain γ in terms of *p* as follows:


$$\begin{array}{l} P(X\geq \ell -\delta )=p,\;\;i.e.,\;\;\;e^{-\gamma (\ell -\delta )}=p, \end{array}$$

thereby giving: γ=−ln   p/ℓ−δ.

Thus, the probability that a sperm is located between ℓ−δ and ℓ is $e^{-\gamma (\ell -\delta )}-e^{-\gamma \ell }$ and the probability that a sperm reaches the implantation site is $[e^{-\gamma (\ell -\delta )}-e^{-\gamma \ell }]\times \delta^{2}/4d\delta \approx \gamma e^{-\gamma \ell }\delta^{2}/4d$_._ The expected number of sperm reaching the implantation site is, therefore, $N\gamma e^{-\gamma \ell }\delta^{2}/4d$.

## RESULTS AND DISCUSSION

Our biophysical modelling #1 indicates that a few thousand sperm cells may reach the implantation site. With *N* = 10^9^, ℓ = 10 cm, *d* = 1 cm and *δ* = 0.02 cm, based on data reported by Hendrickx [4], the number of sperm cells reaching the implantation site is about 10 000. If 50% of the sperm cells remain viable, 5000 viable sperm cells may reach the implantation site. However, the cervical mucus during the mid-luteal phase is less permissive to sperm compared to the peri-ovulation phase. Thus, there may be more sperm wastage during their movement into the uterus.

Modelling #2 assumes an exponential decrease in sperm numbers, resulting in only 1% of the total sperm pool reaching the implantation site. With $N=10^{9},\ell =10\;cm,\delta =0.02\;cm$ and d=1 cm, and choosing *p* to be 0.01, the expected number of hits is about 460. If 50% of sperm cells remain viable, there are 230 hits.

Our modelling for sperm distribution in the uterus suggests that 200–5000 sperm cells may interact with a zona-free implantation-stage blastocyst. Similar data have been reported earlier [5]. Sperm may travel through cervical grooves to the uterine cavity, and hence avoid viscous mucus in the centre of the cervical canal [5]. This process may allow direct interaction of sperm with a zona-free blastocyst at the uterine implantation site.

**Figure 1.**
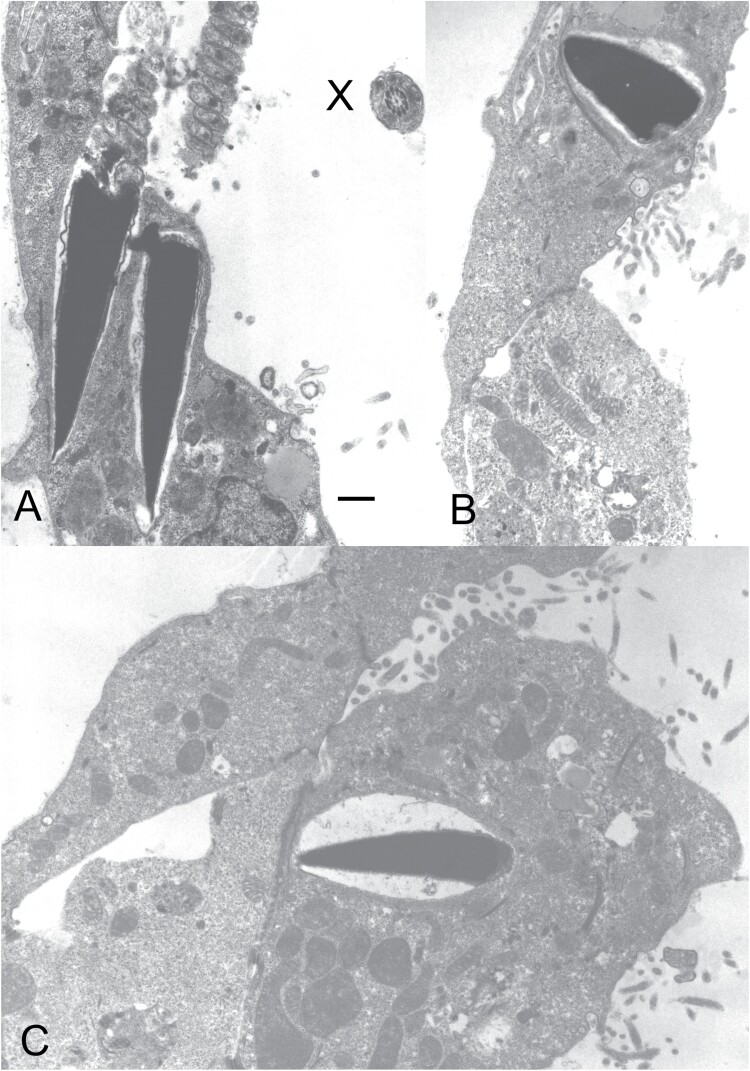
Photomicrographs of transmission electron microscopic images of intruded sperm heads in zona-free blastocysts collected from female rhesus monkeys cohabitating with proven fertile males. (A) Two sperm heads with nuclei bearing highly condensed chromatin and partially disintegrated acrosome membrane along with segments of sperm mid-pieces are seen impregnated within trophectoderm cells of a zona-free blastocyst collected on day 6 after ovulation. The trophoblast cell shows a characteristic large nucleus with dispersed heterochromatin, numerous polyribosomes, apical caveolae, and rough endoplasmic reticulum adjoining lipid droplet and characteristic junctional complexes. A segment of tail end-piece axoneme (X) is also visible. (B) A sperm head bearing condensed chromatin, swollen acrosome and basal plate is seen embedded into a mural trophectoderm cell of another zona-free blastocyst retrieved on day 7 after ovulation. The trophoblast cell bears electron-lucent mitochondria with lamellar cristae, numerous caveolae and short microvilli on its apical surface, lipid droplets rimmed with smooth endoplasmic reticulum, long bundles of filaments, and junctional complexes between adjoining trophoblast cells. (C) Sperm nucleus with highly condensed chromatin present within a large vacuole in a polar trophectoderm cell of a zona-free blastocyst collected on day 8 after ovulation. Numerous long apical microvilli, caveolae, large and small mitochondria showing lamellar cristae, dense cytoplasm with numerous polyribosomes and short filaments distinguish the trophoblast cell from an underlying cell of the inner cell mass that is less dense in structure. Different types of junctional complexes are present between the trophoblast and the cell of inner cell mass, and gap junctions between adjoining trophoblast cells. Bar: 3 µm.

Fig. 1 displays transmission electron microscopic evidence of sperm embedded in the cytoplasm of trophectoderm cells of zona-free blastocysts retrieved during the peri-implantation stage from three rhesus monkeys during their natural pregnancies. These three blastocysts were obtained by uterine flushing on days 6–8 after ovulation from female rhesus monkeys who were cohabitating and had regular vaginal coitus with fertile males resulting in natural conception [1–3]. It is notable that obtaining zona-free intact blastocysts is rather difficult since they are sticky, and tend to promptly adhere to the uterine epithelium. Hence, they are not amenable to retrieval efforts without causing cellular damage [6]. Thus, we cannot comment on the probability of the rate of such sperm insertions in zona-free blastocysts in the natural world. Nevertheless, the sperm–blastocyst interaction in three out of nine blastocysts observed in our study deserves further attention.

This is the first visual evidence of intact sperm heads having entered the trophectoderm of blastocysts, retrieved during the implantation window of rhesus monkeys. It is true that sperm remnants, especially remnants of tails and mid-pieces have earlier been documented in the 1-cell stage to zona-free blastocysts in different mammalian species, and polyspermia is probably more abundant in different mammalian species than originally thought [7–9]. To the best of our knowledge, none of these reports had shown such intact sperm heads and mid-piece intrusion in the trophoblasts of implantation-ready blastocysts as we have reported.

Decades ago, Enders and Schlafke presented a micrograph of ‘a sperm mid-piece in a vacuole in a trophoblast cell’ presumably phagocytosed and also reported, without any microphotographic evidence, about ‘the presence of a sperm head’ in zona-free blastocysts obtained from rhesus monkeys during days 7–8.5 after fertilization [6]. We cannot comment on the report of Enders and Schlafke [6] in which they have reported about the presence of a sperm head in one blastocyst with no supporting image or description. The probability that the observed interaction between sperm and blastocyst occurred from superfluous sperm during fertilization seems to us non-tenable because probabilistically there would be hardly any viable and significantly motile sperm available in the vicinity of implantation-ready blastocysts on day 6–8 after fertilization [10]. We believe that our observed sperm–blastocyst interaction resulted from fresh ejaculation due to the observed integrity of the structures. Nevertheless, a theoretical possibility of an interaction arising from superfluous sperm during fertilization cannot be ruled out.

To our knowledge, there is no significant data in the literature to throw any meaningful light on the issue of sperm from mid-luteal phase coitus entering the trophectoderm of blastocysts during the critical period of implantation. The physiological significance of the present observation that sperm intrudes into the trophectoderm of blastocysts just prior to implantation is only speculative. It leads us to question:

- Can sperm nuclei (containing DNA, RNA and nuclear matrix) entering blastocyst cells act like a Trojan horse and manipulate embryonic expression and female reproduction?
- Is a sperm embedded in blastocyst cells a type of microchimerism? Microchimerism is considered as diploid foreign cells in a genetically distinct host. Here, we observed a haploid sperm of a genetically distinct origin merging with a diploid embryonic cell. It is notable in this regard that in insects ‘unsuccessful’ sperm enter mitosis and form a haploid–diploid chimaera [11].
- Can sperm transmigrate into the epiblast or persist in the trophectoderm, where it may integrate into the placenta, contributing to the higher degree of placenta-confined mosaicism [12]?
- Is it possible that genomically unstable cells, including trisomy cells, may be shunted to the placenta, maintaining a diploid embryo [13]? Keeping genomic stability may be a wasteful process for the transient placental organ.

Sperm interacting with implantation-ready blastocysts can be of paternal or rival origin. Irrespective of the origin, viable sperm may pose challenges to implanting zona-free blastocysts, potentially mediating a selection process for robust and resilient blastocysts. Hence, blastocysts that cannot successfully handle interacting sperm may be more vulnerable to failure during implantation.

This phenomenon might reflect a scenario of parent–offspring conflict [14] and could possibly explain at least in part a reported observation of why frequent coitus around implantation in women may reduce the probability of a positive pregnancy test [15]. However, a high-powered and a better-controlled study conducted subsequently failed to confirm that frequent coitus around implantation indeed affected fecundability in women [16]. If indeed uptake of sperm at the time of implantation confers a disadvantage to embryo development, the fathering male’s sperm may stop implantation of vulnerable blastocysts and allow the menstrual process to set in to initiate another ovulatory cycle. However, this process may not necessarily affect fecundability, in case the blastocysts that were interrupted by sperm were destined to eventually fail to develop regardless of sperm exposure [16].

Non-paternal male (rival) sperm interacting with an implantation-stage blastocyst may cause implantation failure allowing the new male’s genes to be successful in sequelae. Maybe such a phenomenon is a manifestation of reproductive competition at the early stage of embryo development by rival males. It is notable in this regard that social behaviour reflecting reproductive competition is seen in several animal groups including non-human primates [17]. It would be interesting to examine whether there is any mechanism via which we could expect sperm to identify blastocysts that are from the same male versus a rival male. It needs to be stressed though that the possibility that coitus during the implantation window may induce failure of blastocyst implantation, is only a conjecture at this point of time.

Thus, the intrusion of sperm into zona-free blastocysts may initiate a complex adaptive process of interactions between information-inputs from the embryo-self and the environment, possibly affecting the individual embryo’s fate. Possibly, post-ovulatory coitus may have an important influence on the success of implantation and may reflect deep evolutionary significance.

Further studies include (i) DNA typing of sperm to clarify the source of the embedded sperm, (ii) investigations whether the invading sperm persists in the trophectoderm to become only part of the placenta and eventually is rejected leaving the developing embryo untouched, or transmigrates into the epiblast influencing individual embryo’s developmental fate, may reveal important biological facts. Finally, specific knowledge regarding biological consequences of additional sperm in implantation-ready blastocyst cells may have implications for pregnancy success, sperm competition and human health.

## Supplementary Material

eoad043_suppl_Supplementary_Material

## Data Availability

The data that support the findings of this study are available as supplementary data. Additionally, the samples are stored at the Centralized Electron Microscopy Facility, All India Institute of Medical Sciences, New Delhi, India.

## References

[1] Ghosh D , Roy A, Sengupta J et al. Morphological characteristics of preimplantation stage endometrium in the rhesus monkey. Hum Reprod 1993;8:1579–87. 8300810 10.1093/oxfordjournals.humrep.a137895

[2] Ghosh D , Lalitkumar PGL, Wong VJ et al. Preimplantation embryo morphology following early luteal phase anti-nidatory treatment with mifepristone (RU 486) in the rhesus monkey. Hum Reprod 2000;15:180–8. 10611210 10.1093/humrep/15.1.180

[3] Sengupta J , Ghosh D. Blastocyst-endometrium interaction at implantation in the rhesus monkey. J Reprod Immunol 2002;53:227–39. 11730919 10.1016/s0165-0378(01)00091-2

[4] Hendrickx A. Reproduction. In: Embryology of the Baboon. London: University of Chicago Press, 1971, 3–6. ISBN: 0-226-32712-4

[5] Suarez SS , Pacey AA. Sperm transport in the female reproductive tract. Hum Reprod Update 2006;12:23–37. 16272225 10.1093/humupd/dmi047

[6] Enders AC , Schlafke S. Differentiation of the blastocyst of the rhesus monkey. Am J Anat 1981;162:1–21. 7304472 10.1002/aja.1001620102

[7] Sathananthan AH , Ratman SS, Ng SC et al. The sperm centriole: its inheritance, replication and perpetuation in early human embryos. Hum Reprod 1996;11:345–56. 8671223 10.1093/humrep/11.2.345

[8] Wang WH , Day BN, Wu GM. How does polyspermy happen in mammalian oocytes? Microsc Res Tech 2003;61:335–41. 12811738 10.1002/jemt.10346

[9] Sutovsky P. Sperm–oocyte interactions and their implications for bull fertility, with emphasis on the ubiquitin–proteasome system. Animal 2018;12:s121–32. 29477154 10.1017/S1751731118000253 PMC6503950

[10] Ferreira-Poblete A. The probability of conception on different days of the cycle with respect to ovulation: an overview. Adv Contracept 1997;13:83–95. 9288325 10.1023/a:1006527232605

[11] Royer M. Hermaphroditism in insects. Studies on *Icerya purchasi*. In: Reinboth R (ed). Intersexuality in the Animal Kingdom. Berlin: Springer, 1975, 135–45.

[12] West JD , Everett CA. Preimplantation chromosomal mosaics, chimaeras and confined placental mosaicism. Reprod Fertil 2022;3:R66–90. 35514539 10.1530/RAF-21-0095 PMC9066951

[13] Coorens THH , Oliver TRW, Sanghvi R et al. Inherent mosaicism and extensive mutation of human placentas. Nature 2021;592:80–5. 33692543 10.1038/s41586-021-03345-1 PMC7611644

[14] Boddy AM , Fortunato A, Wilson Sayres M et al. Fetal microchimerism and maternal health: a review and evolutionary analysis of cooperation and conflict beyond the womb. Bioessays 2015;37:1106–18. 26316378 10.1002/bies.201500059 PMC4712643

[15] Steiner AZ , Pritchard DA, Young SL et al. Peri-implantation intercourse lowers fecundability. Fertil Steril 2014;102:178–82. 24746744 10.1016/j.fertnstert.2014.03.017 PMC4074557

[16] Stanford JB , Hansen JL, Willis SK et al. Peri-implantation intercourse does not lower fecundability. Hum Reprod 2020;35:2107–12. 32756956 10.1093/humrep/deaa156 PMC8660627

[17] Hrdy SB. Infanticide among animals: a review, classification, and examination of the implications for the reproductive strategies of females. Ethol Sociobiol 1979;1:13–40.

