# Supplementary material for: Sperm intrusion into the implantation-stage blastocyst and its potential biological significance
Source: Evol Med Public Health. 2023 Dec 23;12(1):1–6. doi: 10.1093/emph/eoad043 (PMC10790733; doi:10.1093/emph/eoad043)
Supplement: eoad043_suppl_Supplementary_Material [file eoad043_suppl_supplementary_material.docx]

**Brevia Manuscript**

Sperm intrusion into the implantation-stage blastocyst and its potential biological significance

Running head: Sperm intrusion into implantation-stage blastocyst

Jayasree Sengupta^1aδ^, Thomas Kroneis^2a^, Amy M Boddy^3^, Rahul Roy^4^, Anish Sarkar^4^, Deepayan Sarkar^4^, Debabrata Ghosh^1^*^δ^, Berthold Huppertz^2^*

**Supplementary data**

**Table 1: Availability of data supporting the findings of this study**

| Image^1^ | # of monkey providing material | Mating period (cycle days) | Day of ovulation (cycle day) | Sample collected on (day after ovulation)^2^ |
| --- | --- | --- | --- | --- |
| 1A | 1939 | 8-16 | 10 | 6 |
| 1B | 2307 | 8-16 | 9 | 7 |
| 1C | 2213 | 8-16 | 9 | 8 |

^1^Available with the corresponding authors.

^2^Samples stored at Centralized Electron Microscopy Facility, All India Institute of Medical Sciences. For details see references 1, 2.
